# Methane Production in Dairy Cows Correlates with Rumen Methanogenic and Bacterial Community Structure

**DOI:** 10.3389/fmicb.2017.00226

**Published:** 2017-02-17

**Authors:** Rebecca Danielsson, Johan Dicksved, Li Sun, Horacio Gonda, Bettina Müller, Anna Schnürer, Jan Bertilsson

**Affiliations:** ^1^Department of Animal Nutrition and Management, Swedish University of Agricultural SciencesUppsala, Sweden; ^2^Department of Microbiology, Swedish University of Agricultural SciencesUppsala, Sweden; ^3^Departamento de Producción Animal, Facultad de Ciencias Veterinarias, UNCPBATandil, Argentina

**Keywords:** rumen, CH_4_, microbial community, *Methanobrevibacter*, digestibility

## Abstract

Methane (CH_4_) is produced as an end product from feed fermentation in the rumen. Yield of CH_4_ varies between individuals despite identical feeding conditions. To get a better understanding of factors behind the individual variation, 73 dairy cows given the same feed but differing in CH_4_ emissions were investigated with focus on fiber digestion, fermentation end products and bacterial and archaeal composition. In total 21 cows (12 Holstein, 9 Swedish Red) identified as persistent low, medium or high CH_4_ emitters over a 3 month period were furthermore chosen for analysis of microbial community structure in rumen fluid. This was assessed by sequencing the V4 region of 16S rRNA gene and by quantitative qPCR of targeted *Methanobrevibacter* groups. The results showed a positive correlation between low CH_4_ emitters and higher abundance of *Methanobrevibacter ruminantium* clade. Principal coordinate analysis (PCoA) on operational taxonomic unit (OTU) level of bacteria showed two distinct clusters (*P* < 0.01) that were related to CH_4_ production. One cluster was associated with low CH_4_ production (referred to as cluster L) whereas the other cluster was associated with high CH_4_ production (cluster H) and the medium emitters occurred in both clusters. The differences between clusters were primarily linked to differential abundances of certain OTUs belonging to *Prevotella*. Moreover, several OTUs belonging to the family Succinivibrionaceae were dominant in samples belonging to cluster L. Fermentation pattern of volatile fatty acids showed that proportion of propionate was higher in cluster L, while proportion of butyrate was higher in cluster H. No difference was found in milk production or organic matter digestibility between cows. Cows in cluster L had lower CH_4_/kg energy corrected milk (ECM) compared to cows in cluster H, 8.3 compared to 9.7 g CH_4_/kg ECM, showing that low CH_4_ cows utilized the feed more efficient for milk production which might indicate a more efficient microbial population or host genetic differences that is reflected in bacterial and archaeal (or methanogens) populations.

## Introduction

Ruminants are unique in their ability to utilize feeds rich in cellulose, most likely due to the great diversity of microorganisms that break down feed in the rumen of the host animal. Microorganisms such as bacteria, fungi and protozoa break down complex compounds by hydrolysis to produce volatile fatty acids (VFA), mainly acetate, propionate and butyrate. At the same time, varying amounts of formic acid, hydrogen (H_2_) and carbon dioxide (CO_2_) are produced as end products in fermentation (Hook et al., 2010). Most of the methanogenic archaea in the rumen use H_2_ to reduce CO_2_ to produce methane (CH_4_). This process keeps the partial pressure of H_2_ low, which directs fermentation toward production of less reduced end products including acetate (Moss et al., 2000). Some methanogens in the rumen can also use other substrates for methanogenesis, such as methyl-containing compounds (Hungate, 1967; Liu and Whitman, 2008). The CH_4_ produced is not used by the animal itself, but instead represents an energy loss (2–12% of gross energy) to the atmosphere, mainly by eructation, where it has a negative impact on the climate (Johnson and Johnson, 1995). Therefore, various mitigation strategies have been evaluated with the aim of reducing CH_4_ emissions, including for example using different feed and feedstuffs high in lipids, ionophores, plant compounds such as tannins and enzymes (Beauchemin et al., 2009; Hook et al., 2010; Cieslak et al., 2013). These strategies have produced varying results, but with no significant long-term effect. However, recently, the use of 3-nitrooxypropanol, a compound designed to inhibit the activity of the enzyme responsible for formation of CH_4_ (Duval and Kindermann, 2012), was shown to reduce CH_4_ emissions in dairy cows without any signs of toxic effects on the animal and no or a minor effect on DMI (Reynolds et al., 2014; Hristov et al., 2015; Lopes et al., 2016).

Previous studies have shown a natural variation between individual animals, producing different yields of CH_4_ even for the same feeding conditions (Danielsson et al., 2012; Pinares-Patiño et al., 2013). Studies where CH_4_ has been measured, with both tracer gas and respiration chambers, suggest that there is a repeatable and heritable variation between individuals and thus genetic selection for lower CH_4_ production could be possible (Heimeier, 2010; Pinares-Patiño et al., 2013). However, in order to use genetic selection, it is important to know which factors are important for variation in CH_4_ production and whether these are heritable without negatively affecting productivity. The mechanism causing high/low CH_4_ production in cows is still unclear, but one possible explanation is differences in passage rate due to differences in rumen size (Goopy et al., 2014). Feed is less degraded in a smaller rumen compared with a larger rumen, thus yielding less H_2_ for CH_4_ formation (Goopy et al., 2014). Another source of variation may be linked to differences in community structure of the microbiota in the rumen, a parameter possibly also linked to the difference in passage time.

In the cow rumen, *Methanobrevibacter* seems to be the dominant genus of the archaeal domain (Leahy et al., 2013; Henderson et al., 2015). In our earlier studies, certain groups of *Methanobrevibacter* species (*M. smithii, M. gottschalkii, M. millerae*, and *M. thaueri*), known as the SGMT group (King et al., 2011), were correlated to individuals with higher CH_4_ production but also to feed additive, indicating that specific substrates favor certain *Methanobrevibacter* species (Danielsson et al., 2012, 2014). In a study by Kittelmann et al. (2014), two different types of bacterial communities were linked with low CH_4_ production in sheep.

Due to new molecular techniques, such as next-generation sequencing, knowledge of rumen microbiology has increased in recent years, but the correlation to level of CH_4_ emissions is still not clear. Moreover, it is unclear whether cows producing comparatively lower amounts of CH_4_ have less efficient feed degradation, resulting in lower milk and meat production. To address this question we studied cows, given the same feed, with regard to CH_4_ formation, fiber digestibility, milk production and archaeal- and bacterial community structure. The hypothesis was that cows with similar digestibility and milk production can produce different amounts of CH_4_. These differences are related to individual archaeal- and bacterial community structures in the rumen. The bacterial and archaeal composition in individuals identified as low and high CH_4_ emitters was analyzed by sequence analysis of 16S rRNA gene amplicons using the Illumina MiSeq platform, and quantitative real-time PCR for accurate monitoring of selected taxa.

## Materials and methods

### Animals and experimental design

The study was performed at the Swedish University of Agricultural Sciences, Swedish Livestock Research Center, Lövsta, Uppsala. The experiment was approved by the Uppsala Local Ethics Committee C/124/12. In total, 73 dairy cows (39 Swedish Red (SRB) and 34 Holsteins) in mid-lactation were included in the study. Approximately 45% of the cows were primiparous and 55% were multiparous. Each cow was included in the study from 90 to 180 days post parturition, a period when cows have normally stabilized at high feed intake and milk production. Sampling was performed at three occasions over mid-lactation [days in milk (DIM) ± standard deviation]; the first 133 ± 18 DIM, the second 175 ± 11 DIM, and the third 190 ± 9 DIM.

### Feeds and feeding

The cows were housed in a free-stall barn with an automatic milking system (DeLaval VMS™; DeLaval, Tumba, Sweden). The cows were equipped with neck transponders and had access to separate concentrate blends in two feeding stations for concentrate (DeLaval, Tumba, Sweden) and concentrate was also fed in the milking station. One kilogram of concentrate was accessible each time the cow went for milking in the robot, while the rest of the daily offered concentrate was provided in the concentrate feeding stations. Concentrate was given in pulse doses related to time the cow was in the feeding station, but with a maximum amount of 2 kg at each visit to get total daily amount of concentrate in evenly spread intervals during 24 h. Silage was fed in 20 forage mangers placed on weighing cells, all mangers was available for all cows at all hours. The forage intake was measured by calculating the difference in weight of the manger from when the cow was entering the manger and the weight when the cow was leaving the manger (BioControl, Rakkestad, Norway). Feeding level was based on calculations of the individual nutrient requirements of each cow according to NorFor standards for dairy cows (Volden, 2011). Silage and concentrate were fed separately to all cows throughout the period. The silage was grass-dominated (timothy, fescue, perennial ryegrass) with a small proportion of red clover (<20%), silage chopping length were set to 20 mm. The chemical composition of the feed is shown in Table 1.

**Table 1:** **Nutritional content of feeds, mean (standard deviation) g/kg DM unless otherwise stated**.

**^a^Feed**	***n***	**DM**	**Ash**	**ME (MJ/kg DM)**	**CP**	**NDF**	**AIA**	**pH**	**NH_3_-N, % of DM**
Conc A	15	884 (7)	82.2 (1.6)	14.2	321 (12)	257 (14)	6.1 (0.9)		
Conc B	3	884 (4)	63.9 (0.3)	13.2	193 (2)	239 (12)	5.7 (0.5)		
Conc C	12	888 (6)	65.6 (2.6)	13.2	181 (7)	253 (13)	6.9 (0.8)		
Silage	15	281 (21)	92.9 (7.0)	11.0 (0.2)	136 (10)	478 (22)	25.2 (3.6)	4.1 (0.2)	3.7 (1.1)

*For abbreviations, see text*.

a *Conc, concentrate, trade names (Lantmännen, Malmö, Sweden); Conc A, Unik 82; B, Solid 120; C, Solid 620*.

### Sample collection and analysis

During the three sampling periods, samples of feeds, milk, feces, and rumen fluid representing each period were collected and analyzed. Feed intake and milk production were recorded automatically on a daily basis and CH_4_ production was monitored continuously during milking.

#### Feeds

Samples of silage (~1 L/day) and concentrates (~0.2 L/day) were collected 5d/week during the measurement periods and pooled into one sample per 2-week period. Silage samples were immediately frozen at −20°C. Conventional chemical analyses were performed with standard methods for dry matter (DM), crude protein, neutral detergent fiber (NDF) (NDF, assayed with a heat-stable amylase and expressed exclusive of residual ash) ash, organic matter digestibility of feed stuff *in vitro* (VOS) (from which metabolizable energy (ME) was calculated), as described by Bertilsson and Murphy (2003) and Volden (2011). Determinations of pH and ammonia-N (NH_3_-N), were performed on silage juice. Ammonia-N (NH_3_-N), was analyzed with a flow injection analyze (FIA) technique (Tecator, Application Note, ASN 50-01/92).

#### Digestibility of the feed

Spot samples of feces, about 0.5 L, were collected twice daily (around 8 a.m. and 15 p.m.) on four consecutive days during the measurement periods, frozen and stored at −20°C. The samples were thawed and pooled into one sample per period and cow before analysis. The samples were then freeze-dried, milled and analyzed for DM, ash, NDF, crude protein and acid insoluble ash (AIA). From the total intake of acid insoluble ash and the fecal content of AIA the total amount of feces was calculated (Van Keulen and Young, 1977). Apparent organic matter (OM) digestibility was calculated from estimated intake of organic matter and estimated OM excreted in feces: (OMfeed-OMfaeces)/OMfeed. Apparent digestibility of NDF assayed with a heat-stable amylase and expressed exclusive of residual ash and crude protein was calculated in a similar way.

#### Milk analyses

The cows were milked in the automatic milking system and yield was recorded on a daily basis as kg milk/day. Samples (~20 mL) for analysis of milk composition were obtained morning and evening on two consecutive days at the beginning of each measurement period and stored at +4°C in a refrigerator. Analyses of fat, protein and lactose concentrations in milk were performed by infrared spectroscopy (MilkoScanTM FT120, Foss, Hillerød, Denmark). The analytical values for the separate milking's were weighted according to yield at each milking to a representative sample. Energy-corrected milk (ECM) (4%) was calculated using fat, protein and lactose content of the milk according to Sjaunja et al. (1990).

#### Quantification of CH_4_ emissions

Methane was measured by the method described by Garnsworthy et al. (2012). In brief, CH_4_ concentrations were measured during milking using an infrared CH_4_ analyzer (Guardian Plus; Edinburgh Instruments Ltd., Livingston, UK). The analyzer was calibrated by the use of standard mixtures of CH_4_ in nitrogen. Air was drawn continuously through a polyethylene tube (6 mm inner diameter) at 1 L/min through the instrument by an integral pump between the gas inlet port and the analyzer. The sampling tube was attached on the concentrate trough in the milking robot and the tube were checked daily for blockages. CH_4_ concentration was logged every second on data logger (Simex SRD-99; Simex Sp. z o.o., Gdansk, Poland) and then visualized using logging software (Loggy Soft; Simex Sp. z o.o.). Times of entry to milking station and cow ID were recognized using the VMS management program (DelPro software, version 3.7; DeLaval International AB). Peaks were identified and quantified; raw data from the logger were transformed into values for peak height (maximum minus baseline CH_4_ concentration for each eructation) and integral of peak area (representing total CH_4_ release per eructation). Peaks with a height of less than 200 mg/kg above baseline were discarded. For each milking, mean peak height and integral were calculated, together with peak frequency (eructation rate). Milking occasion with fewer than three recorded peaks were removed from analysis. An index of CH_4_ emission during each milking was calculated as the product of peak frequency and mean peak area. To take into account the dilution of eructed air by the ambient air a factor of dilution was determined and CH_4_ concentrations was adjusted with a dilution factor. A volume of 2.2 L of 1.0% CH_4_ in nitrogen was released into the feed bin. Methane were released at two sites of the trough, at the base and at the center in level with the sampling tube. CH_4_ release was replicated 5 times at each release site and then a mean ratio of CH_4_ concentrations in released and sampled gases was used to convert CH_4_ index to CH_4_ emission rate during milking. CH_4_ was measured throughout the study. To get values for each sampling period, CH_4_ measurement on a 14d basis around the measuring period was used to get an average value for the period.

Based on the total mean CH_4_ production (g/day) for all three periods, the cows were divided into three different groups: high (H), medium (M) and low (L) CH_4_ emitters. If data were missing from one period, or more, the cow was eliminated from the groups.

### Analysis of volatile fatty acids and microbial population

Rumen fluid was collected in mornings around 9–10 a.m. by stomach tubing (Shingfield et al., 2002) for all cows once in each sampling period. In total, one larger sample of 50 mL and three smaller aliquots per cow and period were frozen and stored at −80°C for later analysis of the microbiota and VFA. From each group, 3–4 cows of each of the two breeds (SRB and Holstein) represented in different parities (1st parity or ≥2nd parity), were randomly selected for further analysis of the microbial communities and VFA in rumen fluid. A total of 21 cows were included and from each cow, all three samples (representing three periods) were analyzed. VFA in the rumen contents from the 21 selected cows were determined by HPLC analysis, as described previously by Westerholm et al. (2010).

#### DNA extraction

DNA was extracted from rumen fluid samples in triplicate using 300 μL sample per replicate and the FastDNA® Spin kit for soil (MP Biomedicals, LLC). The extraction step was performed in accordance with the manufacturer's protocol except for an additional purification step to remove PCR-inhibiting component as suggested by the manufacturer, with the procedure for humic acid removal for soil samples (MP Biomedicals, LLC). In brief, samples were washed and re-suspended with a humic acid wash solution, which contained sodium phosphate buffer, MT buffer (provided with the kit) and 5.5 M guanidine thiocyanate. The samples were transferred to SPIN filter, following settling of the binding matrix. In the final step, DNA was eluted by adding 50 μL DNase/Pyrogen-Free water (provided with the kit). DNA concentration was quantified using a Qubit fluorometer (Invitrogen Life Technologies), with a range between 45.7 and 148 ng/μL.

##### Preparation of libraries for amplicon sequencing

16S rRNA amplicon libraries were constructed as triplicates with a two-step PCR. The first PCR simultaneously targeted the V4 region of both bacteria and archaea, using the primers 515′F (GTGBCAGCMGCCGCGGTAA) and 805R (GGACTACHVGGGTWTCTAAT) (Hugerth et al., 2014). The reaction mixtures were set up using Phusion High-Fidelity DNA Polymerase (Thermo Fischer Scientific, Hudson, NH, USA). The reaction mixture contained 5 μL Phusion buffer, 0.5 μL (10 mM) dNTP, 0.75 μL DMSO and 0.25 μL (2 U/μL) Phusion polymerase. The first PCR reaction contained 0.5 μL (10 μM) of each primer, Phusion mix and DNA template. Amplification was performed under the following conditions: initial denaturing step at 98°C for 30 s, 20 cycles of: 10 s at 98°C, 30 s at 60°C, 4 s at 72°C, and a final extension at 72°C for 2 min. The PCR products were checked for size and quality by electrophoresis. Samples were then purified using Agencourt AMPure XP (Becker Coulter, Brea, CA, USA), using a magnetic particle/DNA volume ratio of 0.8:1. The second PCR reaction contained 10 μL purified DNA product, Phusion reaction mix and 1 μL each of the primers 5′-AATGATACGGCGACCACCAGATCTACACX_8_ACACTCTTTCCCTACACGACG-3 and 5′-CAAGCAGAAGACGGCATACGAGATX_8_GTGACTGGAGTTCAGACGTGTGCTCTTCCGATCT-3′, where X_8_ in the primer sequence represented a specific Illumina-compatible barcode. Detailed information about these primers can be found in Hugerth et al. (2014). The barcodes (Eurofins Genomics) were combined, giving a unique combination of barcodes for each sample and thereby allowing for multiplex analysis in the sequencing. The following conditions were used for the second PCR step: initial denaturing at 98°C for 30 s, 8 cycles of 10 s at 98°C, 30 s at 62°C, 5 s at 72°C, and a final extension at 72°C for 2 min. The PCR products were checked by electrophoresis and purified using Agencourt AMPure XP. Each sample was then diluted to the same DNA concentration of 20 nM and pooled to one sample library. The pooled library was sequenced on the MiSeq system (Illumina, Inc., San Diego, Ca, USA) at Science for Life Laboratory/NGI (Solna, Sweden).

##### Sequence analysis

Sequence analysis was performed as described in Müller et al. (2016). In brief, sequences were quality trimmed and trimmed pair end reads were further processed using the QIIME software package, version 1.8 (Caporaso et al., 2010). Sequence data were grouped into operational taxonomic units (OTUs) sharing 97% sequence similarity using an open reference OTU picking strategy. The most abundant sequence in each OTU was selected as representative sequences and further aligned against the Greengenes core set using PyNAST software (Caporaso et al., 2010). Taxonomy was assigned to each OTU using the Ribosomal Database Project (RDP) classifier with a minimum confidence threshold of 80% (Wang et al., 2007). The chimeric sequences were removed by ChimeraSlayer (Haas et al., 2011) and the final OTU table was filtered based on the criteria that the OTU had to be observed in the three replicates to be retained and that one OTU had to contain 57 reads (0.001% of total reads) to be retained. The OTU tables were subsampled (according to the sample containing the smallest set of sequences) to equalize sampling depth. Archaeal sequences were separated from the total sequence set and also assigned using RIM-DB version 13_11_13 (Seedorf et al., 2014), giving more detailed taxonomic information. For further univariate analysis of bacteria, a threshold level at OTUs containing more than 5786 reads (0.1% of total reads) was used. One period for one cow was removed in QIIME because of comparatively fewer reads content in all three replicates (only 0.05% of total reads compared with the other samples). Raw reads have been deposited in SRA at NCBI under accession number PRJNA339907.

##### Real-time PCR

To further investigate the correlation between CH_4_ production and certain group of *Methanobrevibacter* species that was found in Danielsson et al. (2012) and Danielsson et al. (2014), group-specific primers were deigned to target 16S rRNA gene within two *Methanobrevibacter* groups for quantification by real-time qPCR. *Methanobrevibacter* group 1, from now on called *Methanobrevibacter* SGMT, targeted *Methanobrevibacter smithii, Methanobrevibacter gottschalkii, Methanobrevibacter millerae* and *Methanobrevibacter thaueri. Methanobrevibacter* group 2, from now on called *Methanobrevibacter* RO, targeted *Methanobrevibacter ruminantium* and *Methanobrevibacter olleyae*, Primers are presented in Table 2. Group specific primers were designed by using MAFFT v7.017 (Katoh et al., 2002) and Primer3 (Rozen and Skaletsky, 2000) both implemented in Geneious, v6.1.8 (Biomatters, Auckland, New Zealand). Specificity was confirmed both *in silico*, using the BlastN search algorithm provided by the National Library of Medicine, and *in vitro*, by using DNA from pure cultures of *Methanobrevibacter ruminantium* DSM 1093, *Methanobrevibacter olleyae* DSM 16632 (RO group); and *Methanobrevibacter smithii* DSM 861*, Methanobrevibacter gottschalkii* DSM 11977*, Methanobrevibacter millerae* DSM 16643 *and Methanobrevibacter thaueri* DSM 11995 (SGMT group).

**Table 2:** **Primers used for qRT-PCR analysis**.

**Organisms targeted^a^**	**Primer**	**Sequence (5′ to 3′)**	**Product size (bp)**
*Methanobrevibacter SGMT:*	SGMTf	TCCGTAGCCGGTTTAATAAGTCT	~464
	SGMTr	TTCCTCCTATTTATCATAGGCGG	
			
*Methanobrevibacter RO:*	RO55f	GGGGCTAATACCGGATAGATGATT	~636
	ROr	CGACCTCAAGTTCAACAGTATCAC	

a *SGMT, Includes following species of Methanobrevibacter: smithii, gottschalkii, millerae, thaueri*.

*RO, Includes following species of Methanobrevibacter: ruminantium, olleyae*.

For the quantitative analysis, the Bio-Rad iQ5 multicolor real-time PCR detection system was used with IQTM SYBR® Green Supermix (Bio-Rad laboratories, 24 Inc.). All qPCR reactions were set up to a final volume of 20 μL containing the following reagents: IQTM SYBR® Green Supermix 10 μL, forward and reverse primers (10 pmol/μL) 1 μL, DNA template 3 μL and milliQ water 5 μL. The DNA templates prepared from rumen fluid samples were diluted 1:50 and 1:100 prior to analysis. Triplicate DNA samples from each cow and period were analyzed separately. Non-template controls were included in each assay. The program used was as follows: initial temperature 95°C for 7 min, followed by 40 cycles at 95°C for 40 s, 68°C (for RO primer 62°C) for 60 s and 72°C for 40 s. DNA standard curves were prepared from pure cultures of *M. ruminantium* (DSM 1093) for RO group and *M. thaueri* (DSM 11955) for SGMT group, using the group-specific primer set. PCR products were purified and cloned using the pGEMTeasy vector system (Promega, Fitchburg, WI, USA) as recommended by the manufacturer. Chemically competent *Escherichia coli* JM109 cells (Promega) were transformed using the purified PCR products following the manufacturer's instructions. Successful cloning was confirmed by colony PCR. Standard curves consisted of purified plasmid (Qiagen, Plasmid Purification Kit) diluted to 10^8^–10^0^ copy numbers. The specificity of the target PCR product was estimated by melt curve analysis, which consisted of 50 gradual denaturation cycles. The temperature range was set from 55 to 95°C, dwelled 10 s and increased 0.5°C in each cycle. PCR products were also checked by gel electrophoresis. The data generated were collected and analyzed with Bio-Rad iQ5 standard edition optical system software (version 2.0), from which sorted data were exported to Microsoft Excel for further analysis. The efficiency of the RO primer was 94.6% with a slope value of 3.46 and a *R*^2^ value of 0.999. The efficiency of the SGMT primer was 91.8% with a slope value of 3.53 and a *R*^2^ value of 0.987.

### Statistical analysis

Means of individual cow performance parameters were estimated in the MEAN procedure of SAS, which also produced minimum, maximum and standard deviation (SD) values.

Predicted values of CH_4_ production (*Yij, n* = 192) were subjected to the MIXED procedure of SAS (version 9.3; SAS Institute Inc., Cary, NC,5 2008) using the model:

$$\begin{array}{l} Yij={\text{Breed}}_{i}+{\text{Period}}_{j}+{\left(\text{Breed}\times \text{Period}\right)}_{ij}+e_{ijk} \end{array}$$

where the terms are fixed effect of breed (_*i*_ = 2) and period (_*j*_ = 3) and *e*_*ijk*_ is random error.

When analysing for differences between CH_4_ groups (*Yij, n* = 62), the following model was used:

$$\begin{array}{l} Yij={\text{Group}}_{i}+{\text{Period}}_{j}+{\left(\text{Group}\times \text{Period}\right)}_{ij}+e_{ijk} \end{array}$$

where the terms are fixed effect of group (_*i*_ = 3) and period (_*j*_ = 3) and *e*_*ijk*_ is random error.

For both models above: Within every cow, three samples representing each period were used. A spatial power covariance structure, with the sample as the repeated subject and period relative to the sampling as the coordinate for distance between observations, was used in the models to account for the repeated periods within the cow. Least square means were calculated using the LSMEANS/PDIFF option and statistical differences between treatments were determined following the Tukey adjustment (*P* < 0.05).

Principal coordinate analysis (PCoA) was performed in order to find clustering patterns among the samples. The PCoA was based on Bray Curtis distance metrics and analyzed using the PAST software (http://folk.uio.no/ohammer/past/) of clustering patterns was confirmed by a distance-based non parametric MANOVA (Bray Curtis distance, PAST software). For evaluating effects of environmental parameters and differences in OTUs between clusters (*Yij, n* = 59), the following MIXED model was used:

$$\begin{array}{l} Yij={\text{Cluster}}_{i}+{\text{Breed}}_{j}+{\text{Period}}_{k}+{\left(\text{Cluster}\times \text{Period}\right)}_{ij}+e_{ijkl} \end{array}$$

where cow is random effect and the terms are fixed effect of cluster (_*i*_ = 2), Breed (_*j*_ = 2) and period (= 3) and *e*_*ijk*_ is random error. All differences were declared significant at *P* < 0.05. In the analysis of OTUs, false discovery rate procedure (Benjamini and Hochberg, 1995) was applied, which controls for an expected proportion of type I errors.

## Results

For the 73 dairy cows included in the study, average daily dry matter intake was 23.7 ± 2.7 kg/day and ECM yield was 34.6 ± 6.2 kg/day [least square mean ± standard error (s.e.)] (Supplementary Table 1). Methane production in the 73 cows ranged between 282 and 408 g/day, with an average of 318 g/day.

### Individual variation in CH_4_ production between cows

Average daily CH_4_ production by the 73 cows over the three periods was used to select low and high emitters. The selection criteria were that the cow should be persistent low or high CH_4_ emitter over the three measuring periods. Similar numbers of individuals from both breeds and both primiparous and multiparous cows should be included within each group and similar feed intake between groups. Based on these criteria, in total 21 cows were selected for further microbial analyses of rumen fluid. Seven of these cows (3 SRB, 4 Holstein) were low CH_4_ emitters, 6 (2 SRB, 4 Holstein) were high CH_4_ emitters and the other 8 cows (3 SRB, 5 Holstein) were medium CH_4_ emitters. Cows in the medium group were inconsistent in CH_4_ production over the periods and could appear as high in a period and low in another. The CH_4_ production for the low, medium and high groups was 291 ± 7.7, 311 ± 7.0, and 345 ± 8.1 g CH_4_/day [least square mean ± standard error (s.e.)] The difference in CH_4_ production between groups was significant between low and high emitters (*P* < 0.0001) and between medium and high emitters (*P* < 0.05), but not between low and medium emitters (*P* = 0.156). There were no differences in feed intake (kg DM/day) between the low, medium and high emitters (Table 3). For the selected 21 cows, no differences in CH_4_ production were observed between breeds.

**Table 3:** **Intake, production, digestibility of feed and volatile fatty acid (VFA) concentration for high, medium and low emitting cows, ***n*** = number of cows (each cow has three replicates, representing each period)**.

**Item**	**Low (*n* = 6)**	**Medium (*n* = 7)**	**High (*n* = 8)**	**SED^c^**	***P*-value^g^**
Lactation week	24.3^ab^	24.3^a^	23.2^b^	0.48	<0.05
Parity^e^	1.4	1.6	1.5	0.17	ns
Body weight (kg)	652	679	651	27.0	ns
Condition score	3.4	3.3	3.6	0.19	ns
DMI^d^, kg	23.8	26.4	24.2	1.34	ns
Milk yield (kg ECM^e^/d)	36.2	39.1	35.3	3.20	ns
CH_4_,g/day	291^a^	311^ab^	345^c^	10.8	<0.0001
CH_4_/kg DMI	12.4	11.9	14.5	0.92	ns
**Apparent NDF digestibility (g/kg)**					
Neutral detergent fiber (NDF)	64.5	64.0	63.5	1.04	ns
Organic matter (OM)	71.3	70.5	71.0	0.75	ns
Total VFA (mmol/g DM)	67.4	68.9	67.8	4.89	ns
**mol/100 mol**					
Acetate (A)	61.6	61.4	60.4	0.93	ns
Propionate (P)	19.9	18.7	18	1.02	ns
Butyrate (B)	14.8^a^	15.9^ab^	16.9^b^	0.49	<0.05
i-Butyrate	0.74	0.65	0.84	0.118	ns
Valerate	2.99	2.54	3.12	0.280	ns
i-Valerate	0.60	0.46	0.59	0.172	ns
A+B/P	4.03	4.26	4.40	0.301	ns
No. of copies/mL^f^					
M. SGMT	2.5 × 10^7^	1.9 × 10^7^	2.7 × 10^7^	2.1 × 10^7^	ns
M. RO	2.2 × 10^7^^a^	1.4 × 10^7^^ab^	6.8 × 10^6^^b^	5.6 × 10^6^	<0.05

a, b *Different superscript letters within rows indicate that means differ significantly (P < 0.05) between treatments*.

c *SED standard error of difference; highest value chosen*.

d *DMI, Dry matter intake*.

e *ECM, Energy-corrected milk*.

f *SGMT, Includes following species of Methanobrevibacter: Smithii, Gottschalkii, Millerae, Thaueri. RO, Includes following species of Methanobrevibacter: Ruminantium, Olleyae*.

g *ns, not significant*.

e *Parity is considered in two groups (1st parity and ≥2nd parity)*.

### VFA

Total concentration of VFA and proportions of individual fatty acids in the rumen fluid were analyzed to investigate differences in fermentation pattern between groups (Table 3). Differences between the groups were only observed for butyrate, with a significantly lower (*P* = 0.014) proportion in the low CH_4_ emitters group (14.8%) compared with the high CH_4_ emitters group (16.8%).

#### Analysis of microbial composition

The structure of the rumen archaeal and bacterial community in the dairy cows was characterized by sequencing the V4 region of 16S rRNA gene with Illumina MiSeq. After trimming and quality check, a total of 7,354,378 sequences were obtained from 62 (each period per cow is represented by a sample) samples including 118,169 sequences per sample. The number of archaea sequences was 31,341, with an average of 505 sequences per sample (range 153–1190, median 475). The threshold level for bacterial OTU abundance was set to >0.001% and each sample was then subsampled to 93,323 sequences.

##### Archaea

The archaeal community was represented by two different phyla, Euryarchaeota and Crenarchaeota, where Euryarchaeota represented on average 99.9 ± 7.2% (mean ± s.e). Orders were represented by Methanobacteriales (93.8 ± 8.6%), E2 (Thermoplasmata) (6.0 ± 0.5%) and Methanosarcinales (0.1 ± 0.05%). Euryarchaeota was dominated by the genus *Methanobrevibacter*, which represented 88.8 ± 3.60% of the archaea sequences, followed by *Methanosphaera* and unclassified members of the family Methanomassiliicoccaceae, representing 4.8 ± 1.66% and 5.6 ± 2.83% of the archaeal population, respectively. By further use of RIM-DB, certain OTUs taxonomically classified as *Methanobrevibacter* was possible to identify with high similarity (>97%) to the *M. ruminantium* clade and *M. gottschalkii* clade. These clades are represented by very closely related species with 99% identity, where *M. gottschalkii* clade is represented by species of *M. gottschalkii, M. millerae* and *M. thaueri* whereas the *M. ruminantium* clade contain *M. ruminantium* and *M. olleyae* (Seedorf et al., 2015). The *M. ruminantium* clade and *M. gottschalkii* clade represented on average 53 ± 14.0 and 34 ± 12.8% of all archaea sequences detected, respectively.

##### Bacteria

The bacterial population was represented by 18 different phyla, with 17 phyla found across all samples. The bacterial composition was dominated by the Bacteroidetes phylum, representing on average 64 ± 5.9% of all sequences, followed by Firmicutes (24 ± 5.4%), Proteobacteria (5.5 ± 5.8%) and Fibrobacteres (1.1 ± 0.7%). The remaining phyla represented less than 1% of all sequences. At genus level, *Prevotella* dominated, representing on average 48 ± 6.8% of all sequences. Other abundant genera were *Ruminococcus* (3.7 ± 1.10%), *Succiniclasticum* (2.1 ± 1.0%), *Fibrobacter* (1.1 ± 0.7%) and *Butyrivibirio* (1.0 ± 0.3%), as well as unclassified Succinivibrionaceae (4.6 ± 5.7%) and unclassified Lachnospiraceae (3.4 ± 0.9%).

### Differences in microbial community structure between high and low CH_4_ emitter groups

#### Archaea

**Figure 1:**
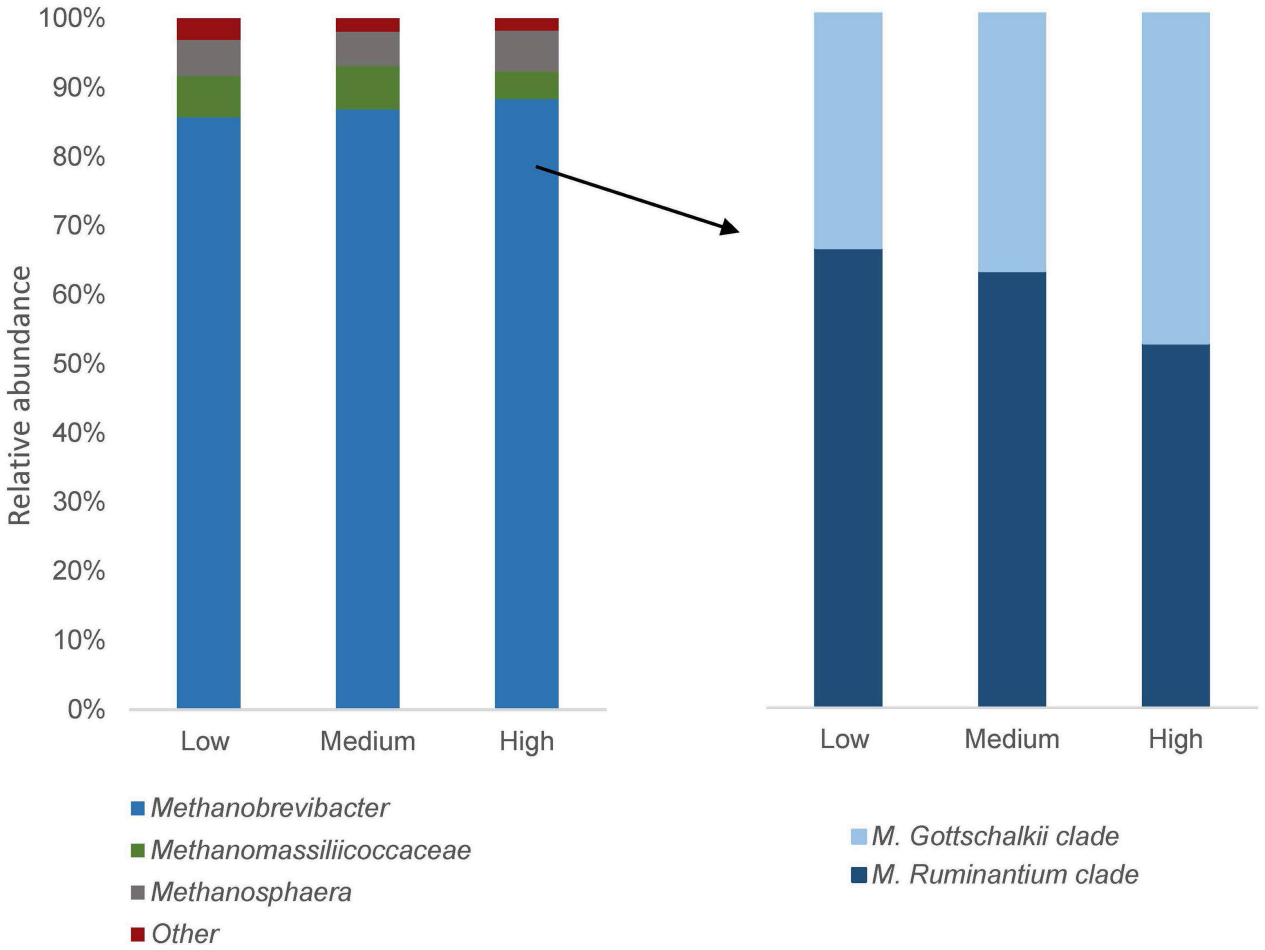
**Relative abundance of archaea in low, medium and high CH_**4**_ emitting groups**. The three bars to the left show genus level (*Methanobrevibacter* in blue, shown by an arrow) and the three bars to the right show the two dominant clades within *Methanobrevibacter* with high similarity (>97%) to *M. ruminantium* and *M. gottschalkii*.

**Figure 2:**
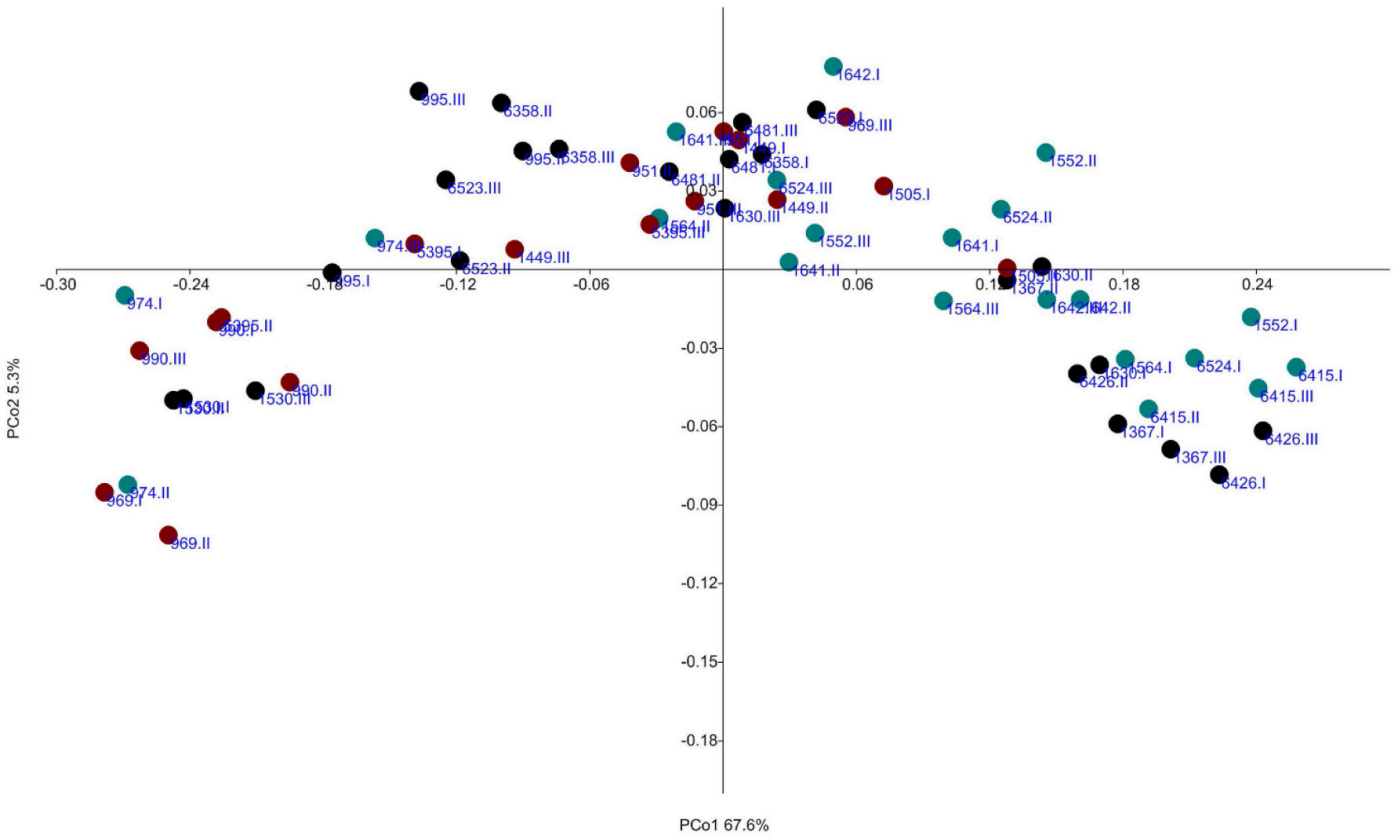
**Principal coordinate analysis (PCoA) showing the relationship of samples based on the archaea operational taxonomic unit (OTU) level**. Colors represent different CH_4_ groups; green, low CH_4_ emitting group; black, medium CH_4_ group; red, high CH_4_ emitting group. Each cow is represented in periods I, II, and III. Principal coordinate (PCo) 1 described 67.6% of the variance and PCo2 5.3%.

Even though there were no significant differences in relative abundance of archaea between high and low emitters, the relative abundance was on average 0.5 ± 0.2% and 0.4 ± 0.2% for high and low CH_4_ group, respectively. Differences between groups were observed at genus level, where unclassified Methanomassiliicoccaceae was 1.5-fold more abundant in low CH_4_ emitters while *Methanobrevibacter* and *Methanosphaera* had almost similar abundance in both groups (Figure 1). At species level, the two clades within *Methanobrevibacter* were compared and a gradual increased ratio of *M. gottschalkii:M. ruminantium* from low to medium to high emitters was observed (Figure 1). *Methanobrevibacter ruminantium* was 1.3-fold more abundant in low emitters and *M. gottschalkii* was 1.5-fold more abundant in high emitters. A PCoA was performed based on Bray Curtis distance metrics on all 21 cows with three samples per cow (each sample representing one period) showed that all low CH_4_ emitters, except one cow, were positioned more at one side of the plot and all high CH_4_ emitters, except one cow, at the other side of the plot (Figure 2). Samples were segregated mainly according to the relative abundance of OTUs belonging to each of the *M. ruminantium* or *M. gottschalkii* clades. Medium CH_4_ emitters were scattered and placed both within the high and low emitting clusters.

Average gene copy numbers retrieved from the qPCR analysis of the two *Methanobrevibacter* clades for RO group were: 1.5 × 10^7^ and for SGMT group 2.3 × 10^7^ 1.5 × 10^7^. A higher copy number of *Methanobrevibacter* RO in low compared with high CH_4_ emitters, 2.3 × 10^7^ ± 3.85 × 10^6^ and 6.8 × 10^6^ ± 4.2 × 10^6^ copies/mL, respectively. No difference between CH_4_ groups was observed for targeted species of *Methanobrevibacter* SGMT (Table 3).

#### Bacteria

**Figure 3:**
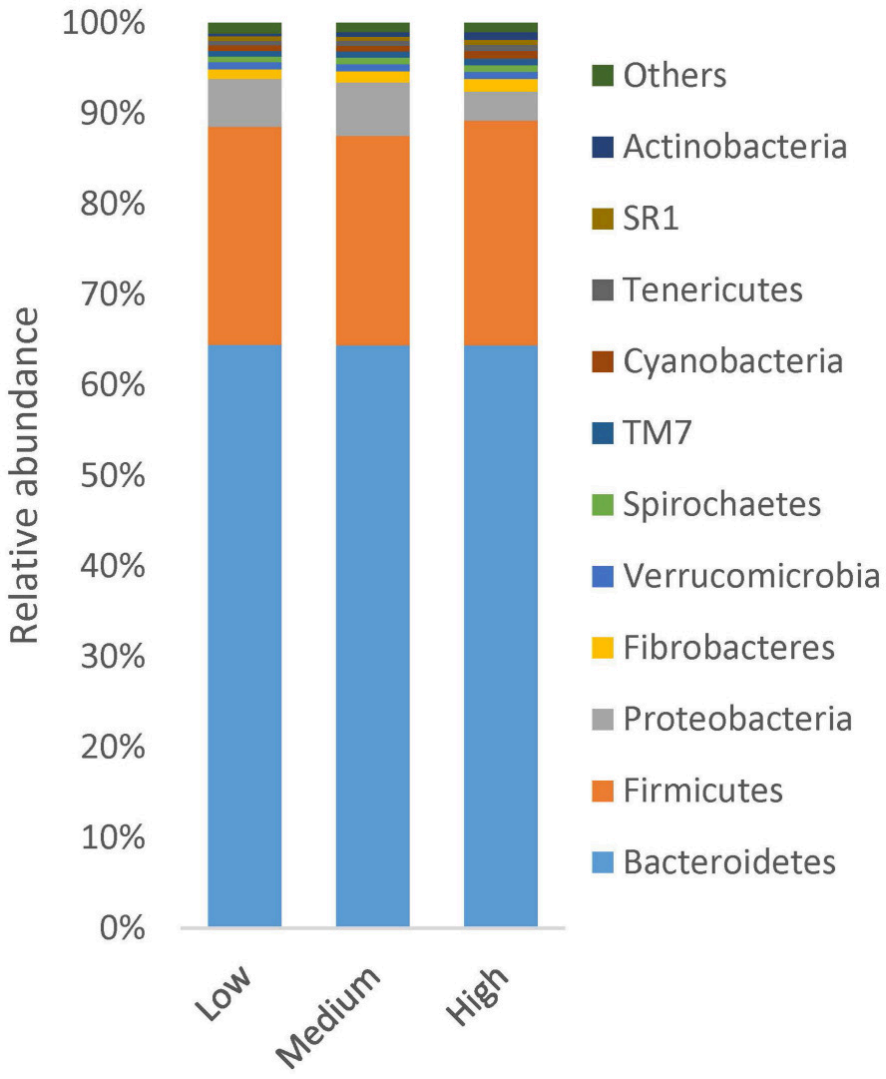
**Relative abundance of bacteria at phylum level in low, medium and high CH_**4**_ emitting group, phyla with lower abundance than 0.001% is assumed as others**.

Analysis using the MIXED procedure revealed no statistically significant differences in the relative abundance of Bacteroidetes or Firmicutes between the CH_4_ groups (Figure 3). Proteobacteria was more abundant in the low CH_4_ group (5.2%) than the high CH_4_ group (3.2%), but this difference was not significant. A significant difference (*P* < 0.05) was only seen for the low abundant phylum Actinobacteria, where the relative abundance was higher in the high CH_4_ emitters group (0.81%) compared with the low CH_4_ emitters (0.32%). At family level, the dominant families were present at similar abundance in both groups of cows, Average relative abundance in the groups of lowest taxa available is presented in Supplementary Figure 1.

**Figure 4:**
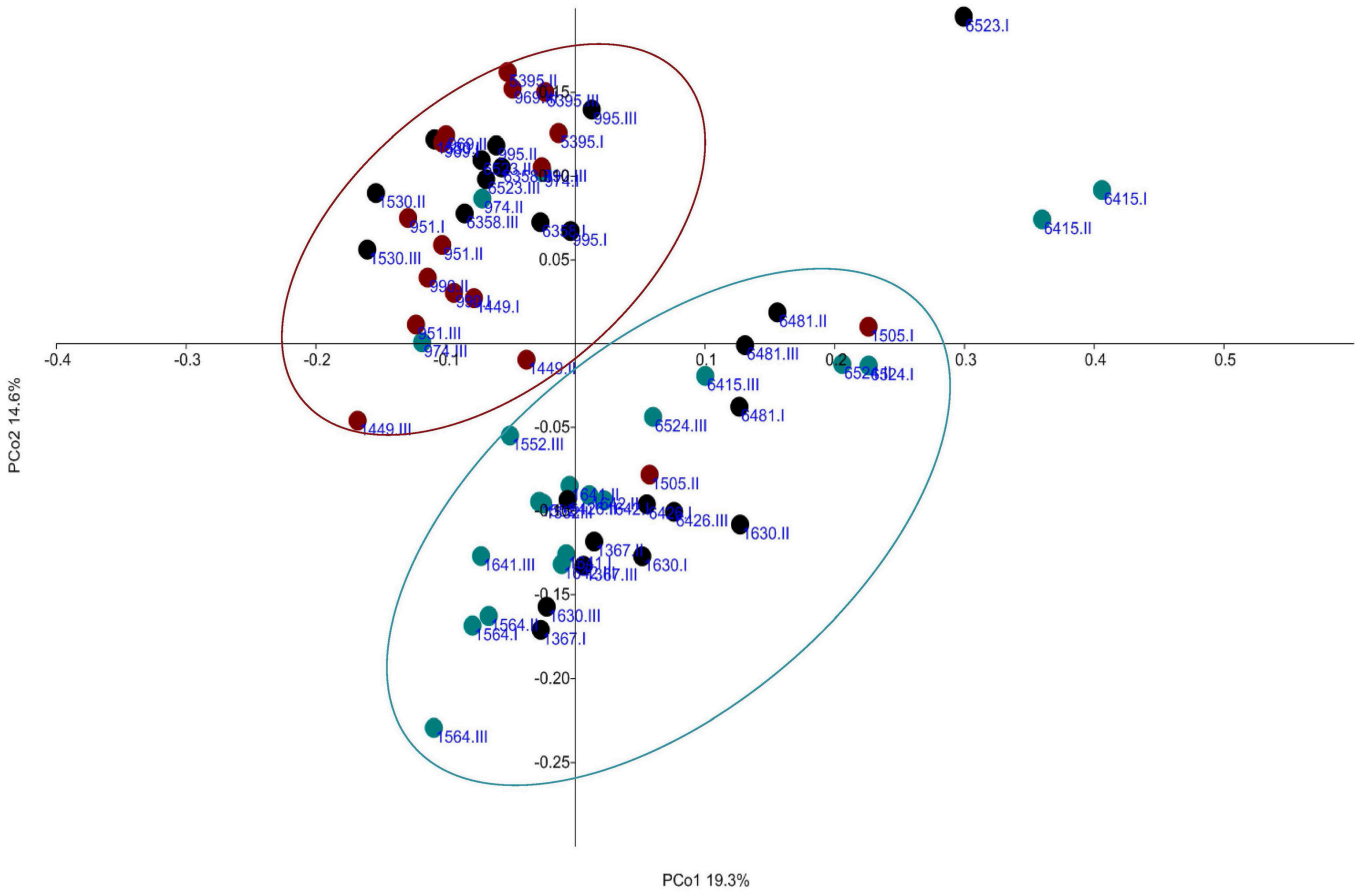
**Principal coordinate analysis (PCoA) defining the relationship between samples based on the bacteria operational taxonomic unit (OTU) level**. Colors represent different CH_4_ groups; green, low CH_4_ emitting group; black, medium group; red, high CH_4_ emitting group. Each cow is represented in periods I, II, and III. Green circle is added to define what further on is called cluster L and red circle cluster H. Three samples were outside the defined clusters. Principal coordinate (PCo) 1 described 19.3% of the variance and PCo2 14.6%.

To further explore whether the bacterial community structure could be linked to the CH_4_ emissions level, a PCoA based on Bray Curtis distance metrics was performed on the bacterial composition at OTU level for all 21 cows (with samples from each period). This analysis exposed two significantly separated clusters (*P* < 0.01), where all low CH_4_ emitters except one (same cow as in archaea plot) were grouped in one cluster (cluster L) and all high CH_4_ emitters except one (same cow as in archaea plot) were grouped in another cluster (cluster H) (Figure 4). Medium CH_4_ emitters were in either one of the two clusters in all three sampling periods. Cluster L included 7 SRB and 4 Holsteins, while cluster H included 2 SRB and 8 Holsteins. To further identify OTUs that contributed to discrimination between the clusters, three outlier samples were removed (Figure 4). The resulting cluster L and cluster H, defined with a green- (cluster L) and a red- (cluster H) circle, was then used for further analyzes. Only the OTUs that had a relative abundance of >0.1% of total reads (in total 154 OTUs) were included in the analysis by MIXED model in SAS. Several of the OTUs that discriminated between the L and H cluster belonged to *Prevotella* (Table 4). Furthermore, an OTU belonging to the family Succinivibrionaceae was present in higher relative abundance in cluster L (2.2%) than cluster H (0.4%). The most abundant OTUs that differed between clusters are presented in Table 4.

**Table 4:** **Mean abundance of operational taxonomic units (OTUs) that was significant different for cluster L and cluster H**.

**OTU taxonomy^a^ OTU reference number**	**Cluster L^b^ (*n* = 11)**	**Cluster H^b^ (*n* = 10)**	**SED^c^**	***P*-value**
**OTUs MORE ABUNDANT IN COWS IN CLUSTER L**				
*Succinivibrionaceae* 807342	1.99	0.44	0.558	0.0078
*Prevotella* 290504	1.67	0.09	0.744	0.0381
*Prevotella* 100265	1.08	0.44	0.097	<0.0001
*Prevotella* 84373	0.88	0.57	0.052	<0.0001
*Prevotella* 2115	0.86	0.68	0.047	0.0003
*Prevotella* 241137	0.82	0.46	0.071	<0.0001
*Prevotella* 268683	0.65	0.26	0.054	<0.0001
*Bacteriodales* 107308	0.48	0.33	0.071	0.0388
*Prevotella* 2093	0.39	0.30	0.029	0.0030
*Prevotella* 205082	0.35	0.26	0.022	<0.0001
*Prevotella* 576319	0.34	0.24	0.030	0.0006
*Paraprevotellaceae* 143138	0.29	0.14	0.038	0.0002
*Ruminococcus* 270733	0.28	0.10	0.031	<0.0001
**OTUs MORE ABUNDANT IN COWS IN CLUSTER H**				
*Prevotella* 86234	0.89	2.00	0.191	<0.0001
*Prevotella* 579901	1.30	1.97	0.138	<0.0001
*Prevotella* 267490	0.68	1.56	0.117	<0.0001
*Succiniclasticum* 2230984	0.88	1.27	0.128	0.0032
*Prevotella* 69910	0.26	1.26	0.097	<0.0001
*Prevotella* 2051	0.67	1.15	0.158	0.0034
*Prevotella* 539926	0.53	1.10	0.073	<0.0001
*Fibrobacter* 262032	0.52	0.83	0.127	0.0176
*Prevotella* 2082	0.46	0.80	0.101	0.0014
*Prevotella* 263905	0.34	0.75	0.054	<0.0001
*Prevotella* 169258	0.41	0.64	0.042	<0.0001
*Bacteriodales* 572735	0.22	0.50	0.055	<0.0001
*Bifidobacteriaceae* 551305	0.01	0.47	0.070	<0.0001

*Number of observations = 59*.

a *Statistical comparisons were only performed on OTUs that had a total abundance of >0.1% of reads. Taxonomy for each OTU is given at highest level for possible classification, each OTU is unique and identified with an OTU number*.

b *Cluster L, associated with low CH_4_ production; Cluster H, associated with high CH_4_ production*.

c *SED, Standard error of difference between cluster*.

### Relationship between cluster and animal parameters

The MIXED model in SAS was used to identify possible relationships between bacterial clusters, different physiological, dietary and production parameters, VFAs and their relationship to the relative abundance of *Methanobrevibacter* clades (*M. ruminantium* and *M. gottschalkii*) and absolute numbers of *Methanobrevibacter* SGMT and *Methanobrevibacter*. RO (Table 5). This analysis showed that the differences between the clusters were primarily related to: CH_4_ production (g/day), gCH_4_/kg ECM, but also the proportions of VFAs, with a relatively higher proportion of propionate in cluster L (19.6%) compared with cluster H (17.1%) (*P* < 0.001). In addition, the proportion of butyrate differed between cluster L (14.7%) and cluster H (17.3%). Differences were also observed for acetate (A) + butyrate (B)/propionate (P) ratio, with a higher ratio in cluster H. The relation to different *Methanobrevibacter* species with different clusters was clear, and similar to differences as those between CH_4_ groups, the difference was related to an increase of *Methanobrevibacter* RO in cluster L compared to cluster H, 2.11 × 10^7^ compared to 6.48 × 10^6^ number of copies/mL, respectively. However, there were no difference in levels of *Methanobrevibacter* SGMT between clusters. Neither were there any differences found in milk production, feed intake or digestibility. Effect of breed within cluster was observed for lactation number, weight, and condition score. Methane production per kilo ECM, lactose, propionate and A+B/P was significant between breeds but with no interaction on cluster (Table 5).

**Table 5:** **Cluster differences according to production, intake, CH_**4**_ emissions, VFA and dominant methanogenic species**.

**Item**	**Cluster L^a^ (*n* = 11)**	**Cluster H^a^ (*n* = 10)**		***P*****-value**		**Interaction**
			**SED^b^**	**Cluster**	**Breed**	**C × B**
Lactation week	23.7	23.4	0.50	ns	ns	ns
Parity^c^	1.6	1.6	0.14	ns	ns	<0.01
Body weight (kg)	660	716	21.3	<0.01	<0.01	<0.001
Condition score	3.3	3.8	0.25	<0.01	<0.0001	<0.01
Milk yield (kg/d)	33.3	33.8	2.08	ns	ns	ns
Milk yield (kg ECM^d^/d)	34.2	33.7	1.81	ns	<0.05	ns
**MILK COMPOSITION (G/KG MILK)**						
Fat	41.6	40.4	1.09	ns	ns	<0.05
Protein	35.6	33.8	1.21	<0.05	ns	ns
Lactose	48.3	46.9	0.50	<0.01	<0.001	ns
**FEED INTAKE, DRY MATTER (KG/D)**						
Dry matter	23.9	24.7	0.88	ns	ns	ns
Organic matter	22.0	22.7	0.81	ns	ns	ns
Crude protein	4.3	4.4	0.16	ns	ns	ns
Neutral detergent fiber	8.5	8.9	0.45	ns	ns	ns
Concentrate	13.8	13.4	0.60	ns	<0.05	ns
Silage	10.6	11.6	0.53	ns	ns	ns
**APPARENT DIET DIGESTIBILITY (G/KG)**						
Dry matter	68.7	69.2	0.76	ns	ns	ns
Organic matter	70.5	71.3	0.71	ns	ns	ns
Neutral detergent fiber	63.7	64.1	1.04	ns	ns	ns
Methane emissions (g/d)	301	321	6.7	<0.001	ns	ns
Methane/kg DMI^e^	12.4	12.9	0.54	ns	ns	ns
Methane/kg ECM	8.3	9.7	0.48	<0.001	<0.001	ns
Total VFA (mmol/g of DM)	68.1	65.9	4.23	ns	ns	ns
**MOL/100 MOL**						
Acetate (A)	61.1	61.8	0.83	ns	ns	ns
Propionate (P)	19.6	17.1	0.63	<0.001	ns	ns
Butyrate (B)	14.7	17.3	0.37	<0.0001	ns	ns
i-Butyrate	0.77	0.73	0.969	ns	ns	ns
i-Valerate	0.51	0.27	0.124	ns	ns	ns
Valerate	3.07	2.80	0.250	ns	ns	ns
A+B/P	3.92	4.72	0.184	<0.0001	<0.05	ns
**ARCHAEA RELATIVE ABUNDANCE (%)**						
*M^f^. gottschalkii clade*	25.4	44.0	2.67	<0.0001	ns	ns
*M. ruminatium clade*	61.8	40.7	2.80	<0.0001	ns	ns

*Number of observations = 59 (each cow has three replicates, representing each period except for two cows that had one or two period samples outside cluster)*.

a *Cluster L, associated with low CH_4_ production; Cluster H, associated with high CH_4_ production*.

b *SED standard error of difference*.

c *Parity is considered in two groups (1st parity and ≥2nd parity)*.

d *ECM, energy-corrected milk*.

e *DMI, dry matter intake*.

f *M, Methanobrevibacter*.

## Discussion

Based on measurements of CH_4_ production from the 73 cows included in this study, it was possible to identify cows that were persistent low and high CH_4_ emitters over a period of 3 months. The method chosen for measurement of CH_4_ production gave lower CH_4_ values than expected, although relative differences between high and low CH_4_ emitters were significant. Analysis of the archaeal and bacterial communities from 6 high, 8 medium, and 7 low CH_4_ emitters revealed that there was a correlation between CH_4_ group and community structure for both the archaea and bacteria.

### Archaea in relation to cows producing low and high emissions of CH_4_

In line with previous studies, no significant difference was seen between the different groups of cows and total abundance of archaea. The main conclusion drawn in previous publications has been that the number of archaea is not essential for the level of CH_4_ production, but rather the metabolic activity of individual methanogenic species is important (Shi et al., 2014). However, in contrast to these results, Wallace et al. (2014, 2015) found a correlation between production of CH_4_ and amount of archaea, based on 16S rRNA gene analysis using qPCR. These authors argue that methanogenesis is the only mechanism of ATP synthesis and therefore it should be a relationship between methane production and methanogens' numbers. The main difference in the present study was found at species level, where the relative abundance of *M. gottschalkii* clade was linked with higher CH_4_ production, and relative abundance of *M. ruminantium* was related to low CH_4_ production. This agrees with our previous finding of an association between the two groups of *Methanobrevibacter* species and CH_4_ production in Swedish dairy cows (Danielsson et al., 2012). A similar linkage between relative abundance of *M. gottschalkii* and high CH_4_ production was found in a study by Shi et al. (2014) investigating the microbiota of sheep. In the present study, specific groups of *Methanobrevibacter* (RO and SGMT) were targeted also by qPCR and the result showed no difference in copy numbers of *Methanobrevibacter* SGMT group between animals with high and low CH_4_ production. Thus, it is likely that the observed difference in relative abundance between animals with high and low CH_4_ production is linked to *M. ruminantium*. In a study by Kittelmann et al. (2013), the relative abundances of the two clades of *M. gottschalkii* and *M. ruminantium* were compared and shown to have a negative relationship (*R*^2^ = 0.51). This means that when the relative abundance of one of the clades was high, that of the other clade was low. A possible explanation for this could be competition for the same substrate, as *Methanobrevibacter* species all are hydrogenotrophs (Leahy et al., 2013) and use hydrogen and/or formate as substrate for their CH_4_ production. Another difference between *M. gottschalkii* and *M. ruminantium* is the presence of genes encoding the key methanogenic enzyme methyl-CoM reductase (Mcr). This enzyme is present in two isomeric forms, McrI and McrII, with the former usually expressed at low H_2_ concentrations and the latter at high H_2_ concentrations (Reeve et al., 1997). M.*ruminantium* M1 does not code for McrII, only for McrI (Leahy et al., 2010), and is thus most likely unable to scavenge hydrogen at higher concentrations. Therefore, different methanogenic species could have an advantage at different H_2_ concentrations and/or respond differently to availability of different CH_4_ substrates (Kittelmann et al., 2014). This study showed two different bacterial clusters in high and low emitting cows and these may have different fermentation patterns, resulting in different amounts of methanogenic substrates, including formate/H_2_, and consequently shaping the methanogenic community.

Indeed Kittelmann et al. (2013) showed that the two different clades seem to have co-occurrence with different species of bacteria, with *M. gottschalkii* clade co-occurring with bacteria from the family Ruminococcaceae and *M. ruminantium* clade linked with bacteria from the family Fibrobacteraceae. Both kinds of bacteria degrade cellulose in the rumen (Kobayashi et al., 2008), but *Ruminococcus* spp. produce large amounts of H_2_, while the two known *Fibrobacter* spp. only produce formate (Rychlik and May, 2000). This co-occurrence seems reasonable as studies on strains of *M. ruminantium* show CH_4_ production from H_2_ together with CO_2_ and from formate (Smith and Hungate, 1958; Leahy et al., 2010), while *M. gottschalkii* grows and produces CH_4_ on H_2_ plus CO_2_, but do not use formate (Miller and Lin, 2002). In contrary to the study by Kittelmann et al. (2013), present study showed a higher relative abundance of *Fibrobacter* spp. in cluster H compared to cluster L, i.e., in the cows having a comparably higher abundance of the *M. gottschalkii* clade. In other study, Kittelmann et al. (2014) identified three different ruminotypes based on the ruminal community structure. Two ruminotypes were associated to low CH_4_ production and one to high CH_4_ production. In these ruminotypes, *Fibrobacter succinogenes* was present at a higher relative abundance in only one of the two low CH_4_ ruminotypes as compared to the high CH_4_ ruminotype. Possibly this differences in results are related to other differences in the ruminal microbial community structure but still it is apparent that the relationship between the methanogenic and bacterial community structure needs further investigations.

### Bacteria in relation to cows producing low and high emissions of CH_4_

Proteobacteria were present at higher abundance in the CH_4_ low emitters compared with the high CH_4_ emitters and were mainly represented by members of the family Succinivibrionaceae. Members of this family produce succinate, an intermediate product in propionate production. Propionate formation is not associated with any hydrogen production, which may explain the comparatively lower CH_4_ production. The correlation between low CH_4_ emitters and Succinivibrionaceae abundance was recently observed for the first time in the cow rumen by Wallace et al. (2015). Members of the Succinivibrionaceae have also been found in wallabies and are suggested to explain their lower CH_4_ production per unit digestible energy intake, which is just 20% of that in cows (Pope et al., 2011).

Analysis of the bacterial community structure revealed two separate clusters that coincided with the low and high CH_4_ emitting groups. In accordance to our findings Kamke et al. (2016) found similar clustering in bacterial community composition in sheep which also was related to high, low and intermediate methane yield emitters. To find an explanation for the apparent difference in clustering and its possible connection to the CH_4_ emission in this study the bacterial composition between these clusters were examined more in detail. This analysis showed that the difference between the clusters was linked to several OTUs that differed in abundance between the two clusters. One of the most prominent differences was related to members of the family Succinivibrionaceae, for which the relative abundance was 5-fold higher in cluster L than cluster H, related to lower CH_4_ production. Actinobacteria in cluster H was represented by OTUs belonging to the families Bifidobacteriaceae and Coriobacteriaceae. *Bifidobacterium* produces lactic and acetic acid. Production of acetic acid instead of more reduced fermentation products is typically associated with increased hydrogen production (Moss et al., 2000), potentially increasing CH_4_ production. Several OTUs that differed in relative abundance between clusters were classified to *Prevotella*, which is usually the main bacterial genus represented in the cow rumen, with many different species observed (Bekele et al., 2010; Kim et al., 2011). Comparison at genus level of *Prevotella* did not reveal any differences between clusters L and H, or between groups of high or low CH_4_ emitters. There were however a difference between clusters at OTU level, i.e., several OTUs of *Prevotella* spp. had a higher abundance in cluster L compared to cluster H and several other OTUs had higher relative abundance in cluster H than cluster L. *Prevotella* spp. produce a variety of extracellular degradative enzymes, which degrade starch and hemicellulose in plant cell walls and also have proteolytic activity, although this varies greatly between *Prevotella* species (Stevenson and Weimer, 2007). Furthermore, it is known to be a great variation in the ability of different *Prevotella* species to utilize certain substrates, a nutritional adaptation that confers an advantage in the rumen environment with different components available through carbohydrate and protein feeds given to the cow (Avguštin et al., 1997; Stevenson and Weimer, 2007). On the other hand, this versatility of substrates makes the role of the *Prevotella* even harder to understand. The potential role of *Prevotella* is difficult to determine in any case, as a large proportion of the population is represented by uncultured species (Bekele et al., 2010). Therefore, we stress the importance of identifying more *Prevotella* spp., in order to understand the functional role of key bacteria in the rumen.

### Relationship between cluster and VFA

Differences in fermentation products such as proportions of butyrate (B) and propionate (P) in relation to total amounts of VFAs were detected. With cluster analysis, no difference was observed for acetate (A) but the ratio A+B/P differed between clusters L and H, indicating that the dominant fermentation pathway differs between bacteria within these two clusters. As mentioned above, formation of acetate and butyrate results in production of additional methanogenic substrates (formate and H_2_), which may explain the increased amount of CH_4_ production in cluster H. Fermentation leading to propionate formation results in less hydrogen being available for CH_4_ production (Moss et al., 2000), which might explain the lower amount of CH_4_ emitted by the cows in cluster L. Similarly, Kittelmann et al. (2014) assumed that proportionally more propionate was present in one of the low CH_4_ emitting ruminotypes in that study.

### Fiber digestion and milk production

Since the mechanism causing high and low CH_4_ emitting cows is unclear, it is important not only to investigate the microbiome but also to examine effects on feed digestion and animal production parameters. Lower CH_4_ production might be related to reduced fiber digestibility, thus also influencing the energy input to the animal. In this study no differences were observed in feed digestibility or milk production in any of the CH_4_ emitting groups. This result was though in contradiction to the study by Pinares-Patiño et al. (2003) where a positive correlation was found between digestion of cellulose and CH_4_ production. In the study by Pinares-Patiño et al. (2003) digestibility was measured by total collection. Choice of method might have an influence on digestibility values, for instance there seem to be a risk of overestimation of digestibility using AIA as internal marker compared to total collection (Lee and Hristov, 2013). Anyhow that risk would be the same for all cows within this study. Furthermore, mean standard deviation for apparent organic matter digestibility was 23 g/kg (average apparent organic matter digestibility value was 706 g/kg, CV = 3.2%) which is close to the 20 g/kg suggested by Van Soest (1994) as being the standard deviation of digestibility determination in carefully conducted experiments. AIA has also shown to provide reliable digestibility estimates in cattle fed grass silage- or hay-based diets (Huhtanen et al., 1994).

In studies on sheep a correlation has been found between CH_4_ production and passage rate of feed particles, amount of liquid and rumen volume, but not with apparent DM digestibility (Goopy et al., 2014). Those authors suggested that no relationship between CH_4_ and digestibility can be found, as the reduced utilization in the rumen is compensated for by increased post-ruminal digestion. This might explain why no differences are observed in whole tract feed digestion, even though there might be differences in microbial populations in the rumen causing differences in digestibility. On the other hand, feed efficiency studies have shown differences in microbial structure between animals in which feed utilization for production of meat or milk also differed (Guan et al., 2008; Zhou et al., 2009; Shabat et al., 2016). In the study by Guan et al. (2008), microbial community profiles from feed-efficient steers were clustered together and differed from those in inefficient steers, showing that the different microbial community related to lowered feed efficiency in rumen was not totally compensated with higher post ruminal digestion. In the recent study by Shabat et al. (2016) feed efficient cows had lower richness in both microbiome- and gene content compared to less feed efficient cows. It was also found that it was possible to predict the animals' feed efficiency phenotype by microbiome genes and species. Propionate to acetate ratio was also higher in efficient animals (Shabat et al., 2016), which strengthen the results of the relationship between microbial community structure and energy available for the cow. Analysis of the different clusters in this study showed a lower CH_4_/kg energy corrected milk (ECM) for cows in cluster L compared to cows in cluster H, 8.3 compared to 9.7 g CH_4_/kg ECM. This results shows that low CH_4_ cows utilized the feed more efficient for milk production which might indicate a more efficient microbial population or some host genetic differences that have an impact on the microbial community structure.

## Conclusions

The majority of the investigated cows were consistently low or high CH_4_ emitters over the studied period, 3 months, but no effect were seen on fiber digestion or milk production. The cows were grouped into two different clusters that differed in abundance of the *Methanobrevibacter* clades *M. ruminantium* and *M. gottschalkii*. Higher relative abundance of *M*. *ruminantium* and also copy numbers of the targeted group *Methanobrevibacter RO* were associated with the low CH_4_ emitting group. The bacterial communities also differed between high and low emitting cows, possibly the reason for varying methanogen communities. Furthermore, the results from this study suggest that differences in the microbiota among individuals is linked with difference in the degree of CH_4_ production.

## Author contributions

RD: Planning, design, lab work, analysis at lab and analysis data and interpreting all results, and writing. JD: Planning, design, analysis of sequence data and interpreting result, and reading manuscript. LS: Processing of sequencing data, writing processing procedure part, submission of sequences and reading manuscript. HG: Planning, sampling in barn, some interpretation, and reading manuscript. BM: Design of primers for qPCR, writing design procedure, interpreting result, and reading manuscript. AS: Planning, design, interpreting results, and reading manuscript. JB: Planning, design, sampling in barn, interpreting results, and reading manuscript.

### Conflict of interest statement

The authors declare that the research was conducted in the absence of any commercial or financial relationships that could be construed as a potential conflict of interest.

## Supplementary material

Supplementary Figure 1 **Relative abundance of bacteria identified at lowest taxa level in low, medium, and high CH_**4**_ emitting group, taxa with lower abundance than 1% has been summarized**. Characters within parenthesis shows which phylum the taxa belongs to; B, Bacteroidetes; P, Proteobacteria; F, Firmicutes; Fib., Fibrobacteres.

Supplementary Table 1 **Means of individual cow performance; production, intake and methane emissions**. Range (min and max) and standard deviation (SD). Number of observations = 192.

## Abbreviations

AIA: Acid insoluble ash
DIM: Days in milk
DM: Dry matter
ECM: Energy corrected milk
ME: Metabolizable energy
NDF: Neutral detergent fiber
SRB: Swedish red Breed.
