# Supplementary figures and images for: Methane Production in Dairy Cows Correlates with Rumen Methanogenic and Bacterial Community Structure
Source: Front Microbiol. 2017 Feb 17;8:226. doi: 10.3389/fmicb.2017.00226 (PMC5313486; doi:10.3389/fmicb.2017.00226)

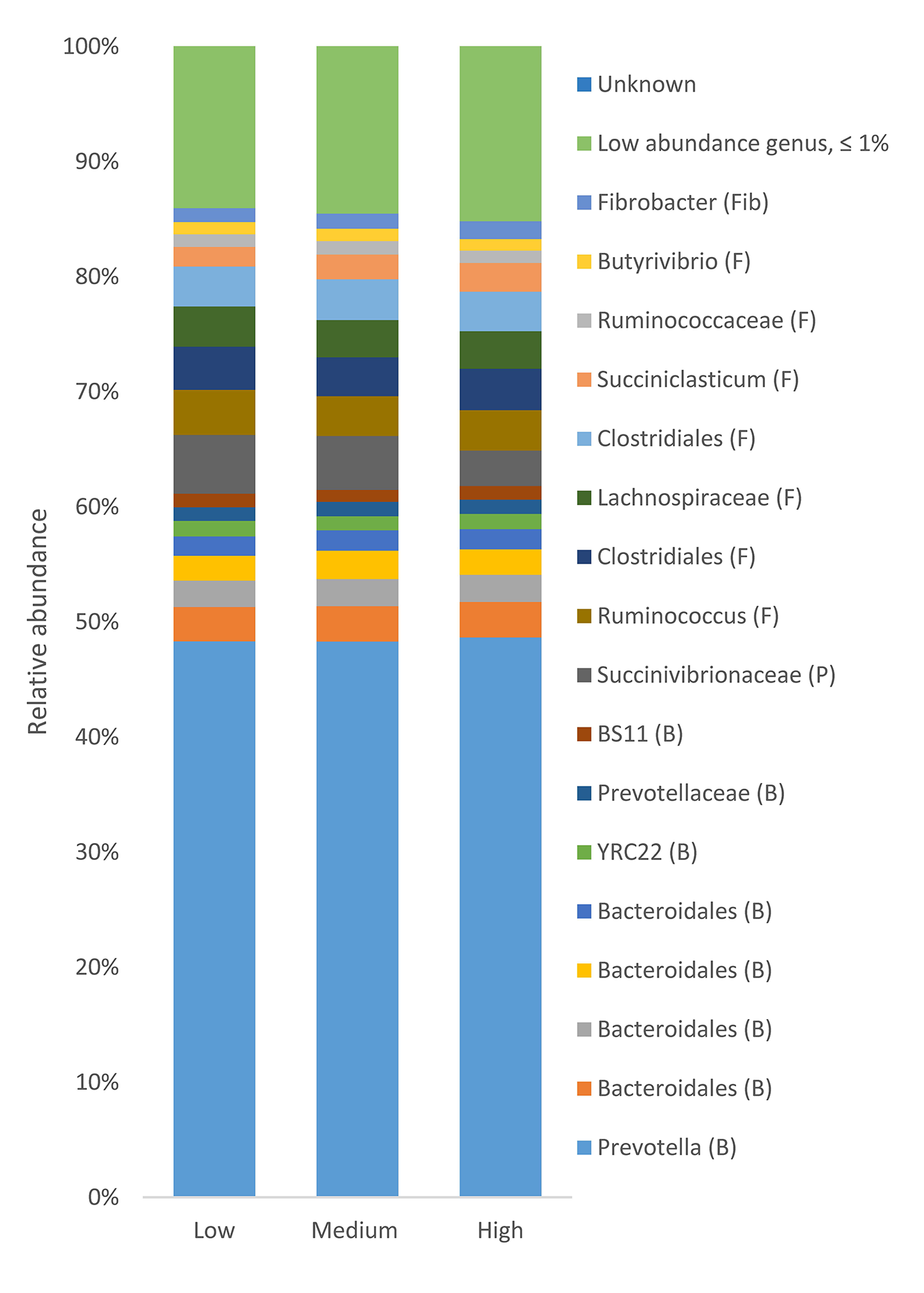

Supplement: Supplementary Figure 1 — Relative abundance of bacteria identified at lowest taxa level in low, medium, and high CH4 emitting group, taxa with lower abundance than 1% has been summarized. Characters within parenthesis shows which phylum the taxa belongs to; B, Bacteroidetes; P, Proteobacteria; F, Firmicutes; Fib., Fibrobacteres. [file Image1.TIF]
