# Supplementary material for: Methane Production in Dairy Cows Correlates with Rumen Methanogenic and Bacterial Community Structure
Source: Front Microbiol. 2017 Feb 17;8:226. doi: 10.3389/fmicb.2017.00226 (PMC5313486; doi:10.3389/fmicb.2017.00226)
Supplement: Supplementary Table 1 — Means of individual cow performance; production, intake and methane emissions. Range (min and max) and standard deviation (SD). Number of observations = 192. [file Table1.DOCX]

**Supplementary Table S1.** Means of individual cow performance; production, intake and methane emissions. Range (min and max) and standard deviation (SD). Number of observations =192

| Item | Mean | Min | Max | SD |
| --- | --- | --- | --- | --- |
| Lactation week | 23 | 14 | 31 | 4 |
| Parity | 2 | 1 | 8 | 2 |
| Body weight (kg) | 646 | 511 | 913 | 72 |
| Condition score | 3.4 | 2.5 | 5.0 | 0.5 |
|  |  |  |  |  |
| Milk yield (kg/d) | 34.6 | 13.9 | 55.2 | 6.9 |
| Milk yield (kg ECM^a^/d) | 34.6 | 14.5 | 54.8 | 6.2 |
| Milk composition (g/kg milk) |  |  |  |  |
| Fat | 41.6 | 29.9 | 53.5 | 4.6 |
| Protein | 34.3 | 28.9 | 40.7 | 2.4 |
| Lactose | 48.0 | 41.8 | 50.9 | 1.6 |
|  |  |  |  |  |
| Feed intake (kg/d) |  |  |  |  |
| Dry matter | 23.7 | 16.3 | 31.2 | 2.7 |
| Organic matter | 21.8 | 15.0 | 28.8 | 2.5 |
| Crude protein | 4.22 | 2.55 | 5.63 | 0.56 |
| Neutral detergent fibre | 13.2 | 6.09 | 18.6 | 2.31 |
| Proportion of concentrate (per kg DM intake) | 0.56 | 0.36 | 0.70 | 0.07 |
|  |  |  |  |  |
| Apparent diet digestibility (g/kg) |  |  |  |  |
| Dry matter | 683 | 613 | 761 | 23 |
| Organic matter | 706 | 643 | 775 | 2 |
| Neutral detergent fibre | 637 | 533 | 743 | 42 |
|  |  |  |  |  |
| Methane emissions (g/d) | 316 | 282 | 408 | 29 |
| Methane/kg DMI^b^ | 13.4 | 9.6 | 20.2 | 2.5 |

^a^ECM, energy-corrected milk

^b^DMI, dry matter intake
